# Quantitative Analysis and Visualization of the Interaction Between Intestinal Microbiota and Type 1 Diabetes in Children Based on Multi-Databases

**DOI:** 10.3389/fped.2021.752250

**Published:** 2021-12-15

**Authors:** Mingyi Zhao, Shaokang Xu, María José Cavagnaro, Wei Zhang, Jian Shi

**Affiliations:** ^1^Department of Pediatric, The Third Xiangya Hospital, Central South University, Changsha, China; ^2^Xiangya School of Medicine, Central South University, Changsha, China; ^3^College of Medicine-Phoenix, University of Arizona, Phoenix, AZ, United States; ^4^Department of Spine Surgery, The Third Xiangya Hospital, Central South University, Changsha, China; ^5^Department of Hematology and Critical Care Medicine, The Third Xiangya Hospital, Central South University, Changsha, China

**Keywords:** intestinal microbiota, childhood autoimmune diseases, type 1 diabetes, quantitative analysis and visualization, multi-databases, pediatric microbiome

## Abstract

**Background:** As an important autoimmune disease, type 1 diabetes (T1D) is often diagnosed in children, but due to the complexity of the etiology of diabetes and many other factors, the disease pathogenesis of diabetes is still unclear. The intestinal microbiota has been proved to have close relationships with T1D in recent years, which is one of the most important molecular bases of pathogenesis and prognosis factors for T1D. Using the multi-omics and multicenter sample analysis method, a number of intestinal microbiota in T1D have been discovered and explained, which has provided comprehensive and rich information. However, how to find more useful information and get an intuitive understanding that people need conveniently in the huge data sea has become the focus of attention. Therefore, quantitative analysis and visualization of the interaction between intestinal microbiota and T1D in children are urgently needed.

**Methods:** We retrieved the detailed original data from the National Center for Biotechnology Information, GMREPO, and gutMEGA databases and other authoritative multiple projects with related research; the ranking of intestinal microbiota abundance from healthy people, overall T1D patients, and T1D in children (0–18 years old) were detailed analyzed, classified, and visualized.

**Results:** A total of 515 bacterial species and 161 related genera were fully analyzed. Also, *Prevotella copri* was led by 21.25% average abundance, followed by *Clostridium tertium* of 10.39% in all-cross T1D patients. For children with T1D, *Bacteroides vulgatus* has high abundance in all age periods, whereas the abundance of each intestinal microbiota was more uniform in female samples, with the ranking from high to low as *Bacteroides dorei* 9.56%, *P. copri* 9.53%, *Streptococcus pasteurianus* 8.15%, and *C. tertium* 7.53%, whereas in male samples, *P. copri* was accounted for the largest by 22.72%. The interaction between intestinal microbiota and comparison between healthy people and children with T1D was also detailed analyzed.

**Conclusions:** This study provides a new method and comprehensive perspectives for the evaluation of the interaction between intestinal microbiota and T1D in children. A set of useful information of intestinal microbiota with its internal interaction and connections has been presented, which could be a compact, immediate, and practical scientific reference for further molecular biological and clinical translational research of T1D in children.

## Introduction

As one of the most common chronic autoimmune diseases, type 1 diabetes (T1D) is characterized by the destructive changes of insulin-producing β cells, which leads to hyperglycemia and deficiency of insulin (1–3). Islet cell antibody, insulin antibody, and glutamic acid decarboxylase antibody can be found in T1D. As for idiopathic T1D, there is no evidence of autoimmune antibodies, but the islet function is also lacking (4). T1D may occur at any age but predominantly affects children and adolescents, which carries serious health risks and considerable social burdens (5). The fundamental management of T1D remains a tremendous challenge, demanding strict blood glucose monitoring, carbohydrates restricting, and insulin and its analogs related therapying (6–8). Various factors are involved in the development of T1D, including diet, genome, and gut microbiota, but due to the complexity of the etiology of diabetes and many factors, the disease pathogenesis of diabetes is unclear, so the treatment of T1D also cannot fundamentally be solved. Proper understanding of the disease pathogenesis may help in developing new therapeutic strategies that improve the control and prevent the complications associated with T1D.

Currently, intestinal microbiota has been proved to have close relationships with numerous autoimmune diseases (AIDs), including Kawasaki disease, juvenile idiopathic arthritis, Henoch–Schonlein purpura, multiple sclerosis, T1D, etc. (9–15). Especially in T1D, intestinal microbiota plays an important role in the maturation of the immune system, which has been widely studied. Among these research subsets, factors such as diet and hormones have played a part in the progression of T1D results in the relative influencing factors on the composition of intestinal microbiota and its connectivity and stability (16, 17). Besides, the host immune system and related whole and local immune state could be altered by intestinal microbiota, therefore, plays an important role in the generation, progress, and prognosis of T1D (18–20).

Recent evidence shows that intestinal microbiota is highly associated with the pathogenesis of insulin dysfunction and T1D. Furthermore, over the last few years, in the wake of multi-omics and high-throughput sequencing techniques, the understanding of T1D progression was deepened, and the capability to the practical clinical application of disease knowledge was improved, and research to the necessity of information for physicians and scientists was enhanced. Currently, several integrated multi-omics of the human intestinal microbiota have been done (21–23). However, due to the complexity of T1D-related intestinal microbiota, which includes a number of subsystems and huge and complicated data, it was hard for clinical physicians and researchers to obtain convenient, direct viewing, and highly effective information. Thus, in this study, a series of quantitative analyses and visualization of comprehensive data in the intestinal microbiota species abundance and other important information with T1D in children from multi-databases were done, which hope to shed more light on the intuitive display with clear explanation, concise presentation, and stimulate thought to the research field.

## Materials and Methods

### Data Mining and Acquisition

We retrieved the detailed data from the National Center for Biotechnology Information (NCBI) and the GMREPO databases; we found the original big data of intestinal microbiota of patients with type 1 urinary disease aged 0–18 years (infancy to adolescence, patients with other diseases were excluded) and obtained the original data of all samples through NCBI data source link (Original BioProject: PRJNA289586/PRJNA445932/PRJNA387903) (23–29). In addition, we searched another authoritative database, gutMEGA, for additional data from related research (30). The relative abundance of intestinal flora in all patients with T1D and healthy people aged 0–18 years were also obtained from GMREPO.

### Original Data Analysis

The R programming language (R4.0.5 for Windows 64 bit) was used for data cleaning and pretreatment according to the standard format and notation of different databases. Doubtful, incomplete, or unknown data removing process and source data integration were also done by the R programming language. After the average values of all kinds of bacteria were calculated, the Fuzzy C means clustering and correlation analysis was carried out.

### Statistical Analysis

Statistical analysis was performed using the statistical software IBM SPSS 19.0, and the measurement data were analyzed by mean ± standard deviation.

## Results

### Abundance Ranking of Type 1 Diabetes-Related Intestinal Microbiota

Original data of intestinal microbiota abundance were obtained from GMREPO and sorted according to the average bacteria abundance. Table 1 shows the top 30 species with relative abundance, whereas the top 10 genera of intestinal microbiota with T1D are shown in Table 2.

**Table 1:** Ranking of top 30 intestinal microbiota with T1D (Species).

**Scientific Name**	**NCBI Taxon ID**	**Mean Relative Abundance (%)**
*Prevotella copri*	165179	21.25
*Clostridium tertium*	1559	10.39
*Phascolarctobacterium succinatutens*	626940	8.59
*Bacteroides eggerthii*	28111	7.57
*Bacteroides uniformis*	820	7.08
*Streptococcus pasteurianus*	197614	6.90
*Dialister succinatiphilus*	487173	5.61
*Bacteroides dorei*	357276	4.99
*Faecalibacterium prausnitzii*	853	4.67
*Ruminococcus bromii*	40518	4.19
*Clostridium sp. L2-50*	411489	4.15
*Eubacterium rectale*	39491	4.12
*Escherichia coli*	562	3.31
*Eubacterium eligens*	39485	2.97
*Clostridium septicum*	1504	2.92
*Eubacterium siraeum*	39492	2.78
*Bacteroides vulgatus*	821	2.75
*Catenibacterium mitsuokai*	100886	2.66
*Prevotella stercorea*	363265	2.44
*Methanosphaera stadtmanae*	2317	2.39
*Bacteroides sp. 2_1_22*	469588	2.23
*Ruminococcus sp. 5_1_39BFAA*	457412	2.22
*Bacteroides ovatus*	28116	2.12
*Ruminococcus torques*	33039	2.11
*Alistipes putredinis*	28117	2.04
*Lactobacillus floricola*	679249	2.04
*Barnesiella intestinihominis*	487174	1.96
*Clostridium ventriculi*	1267	1.78
*Akkermansia muciniphila*	239935	1.66
*Holdemanella biformis*	1735	1.66

**Table 2:** Ranking of top 10 intestinal microbiota with T1D (Genus).

**Scientific Name**	**NCBI Taxon ID**	**Mean Relative Abundance (%)**
*Prevotella*	838	21.99
*Bacteroides*	816	20.58
*Eubacterium*	1730	9.91
*Phascolarctobacterium*	33024	8.59
*Ruminococcus*	1263	5.92
*Subdoligranulum*	292632	5.64
*Alistipes*	239759	5.10
*Clostridium*	1485	5.05
*Faecalibacterium*	216851	4.67
*Bifidobacterium*	1678	3.51

### Multi-Sample-Based Compound Quantitative Statistics

**Figure 1:**
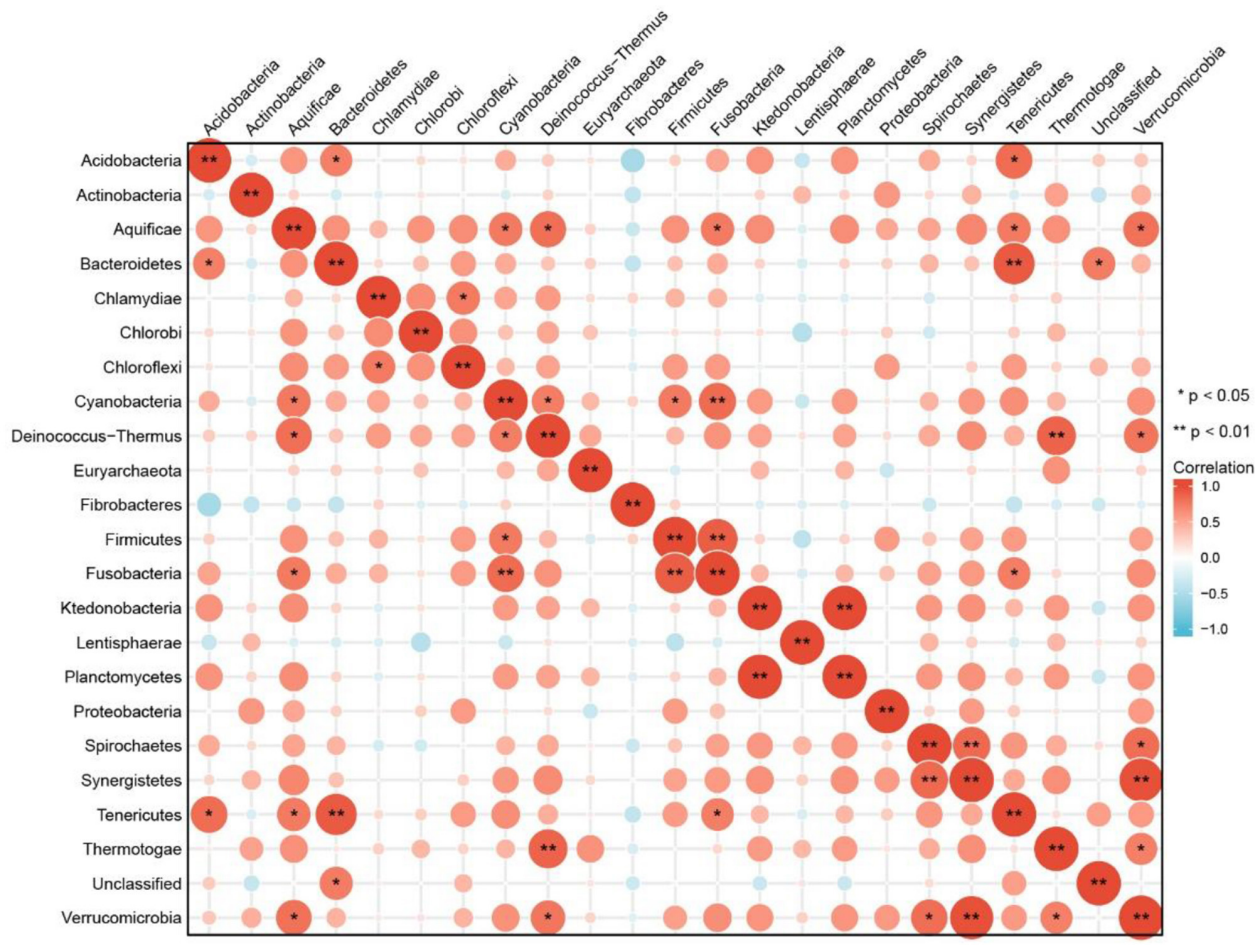
Relativity analysis of intestinal microbiota in T1D based on multi-samples.

**Figure 2:**
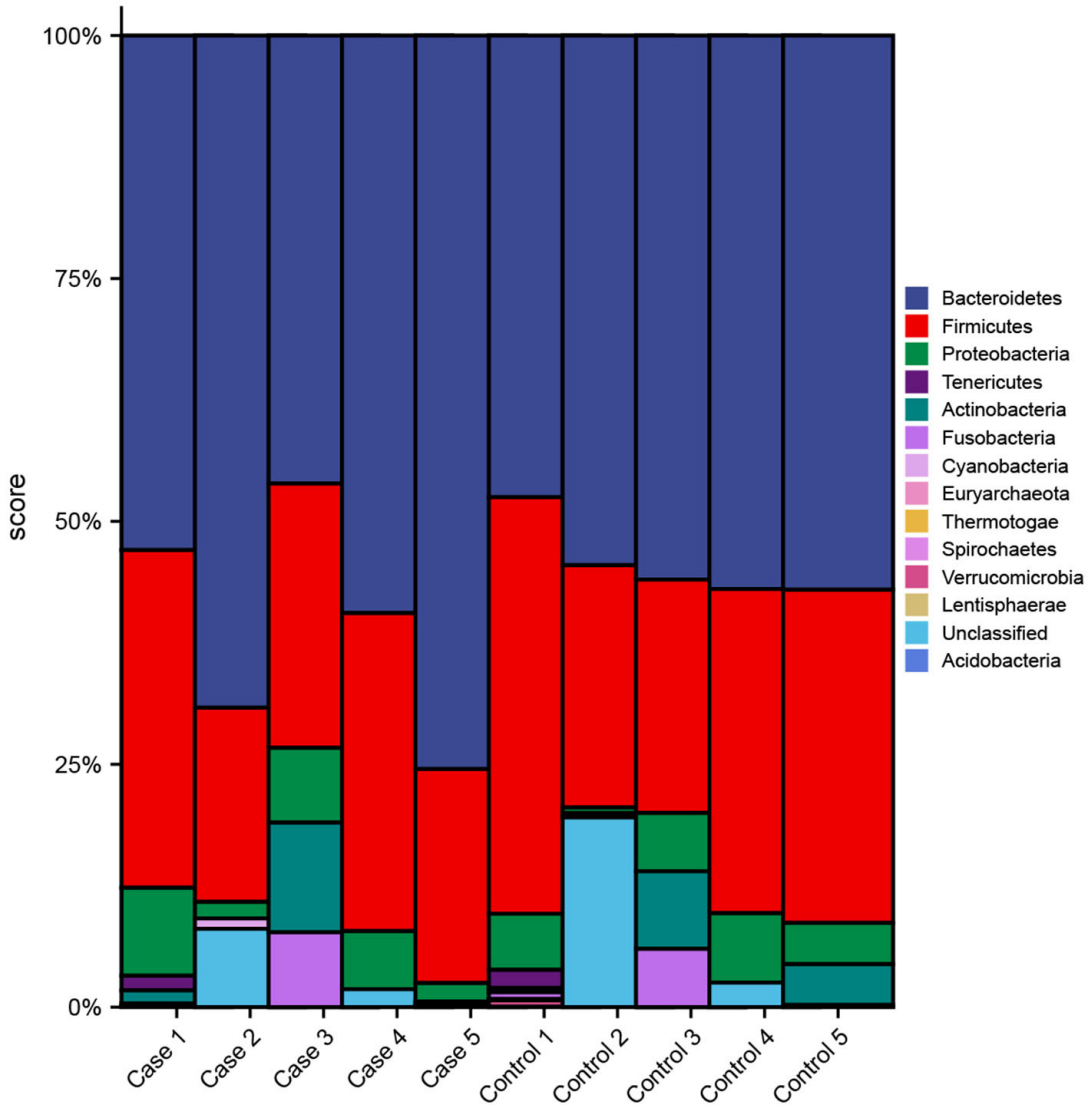
Difference analysis of intestinal microbiota in T1D based on multi-samples.

Then, a further analysis on multi-samples from GMREPO, gutMEGA, and related research (23–34) for T1D was done. Spearman correlation analysis of 23 different phyla of intestinal microbiota was also carried out (Figure 1). *Fusobacteria* was positively correlated with *Cyanobacteria*, whereas *Fusobacteria* was positively correlated with *Firmicutes*. On the whole, only a few strains have a negative correlation, and their significance is not so strong. In addition, we also analyzed the difference in flora abundance between the control and case groups (Figure 2). The results also revealed that *Bacteroidetes* have a higher relative abundance in case groups, whereas *Firmicutes* have a higher relative abundance in the healthy group.

### Data Visualization by Integrating Multi-Projects

**Figure 3:**
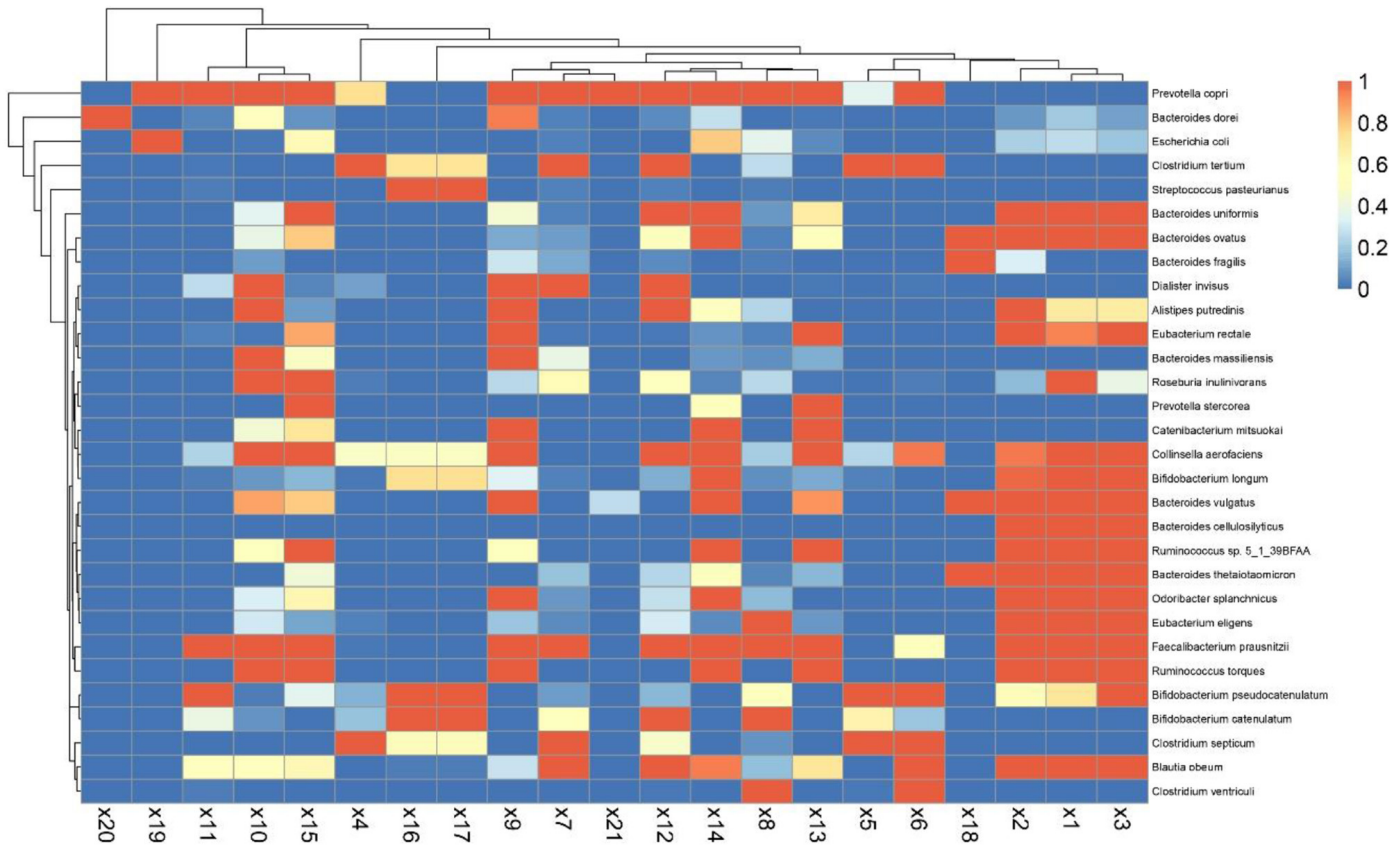
Visualization of mean intestinal microbiota abundance in children with T1.

**Figure 4:**
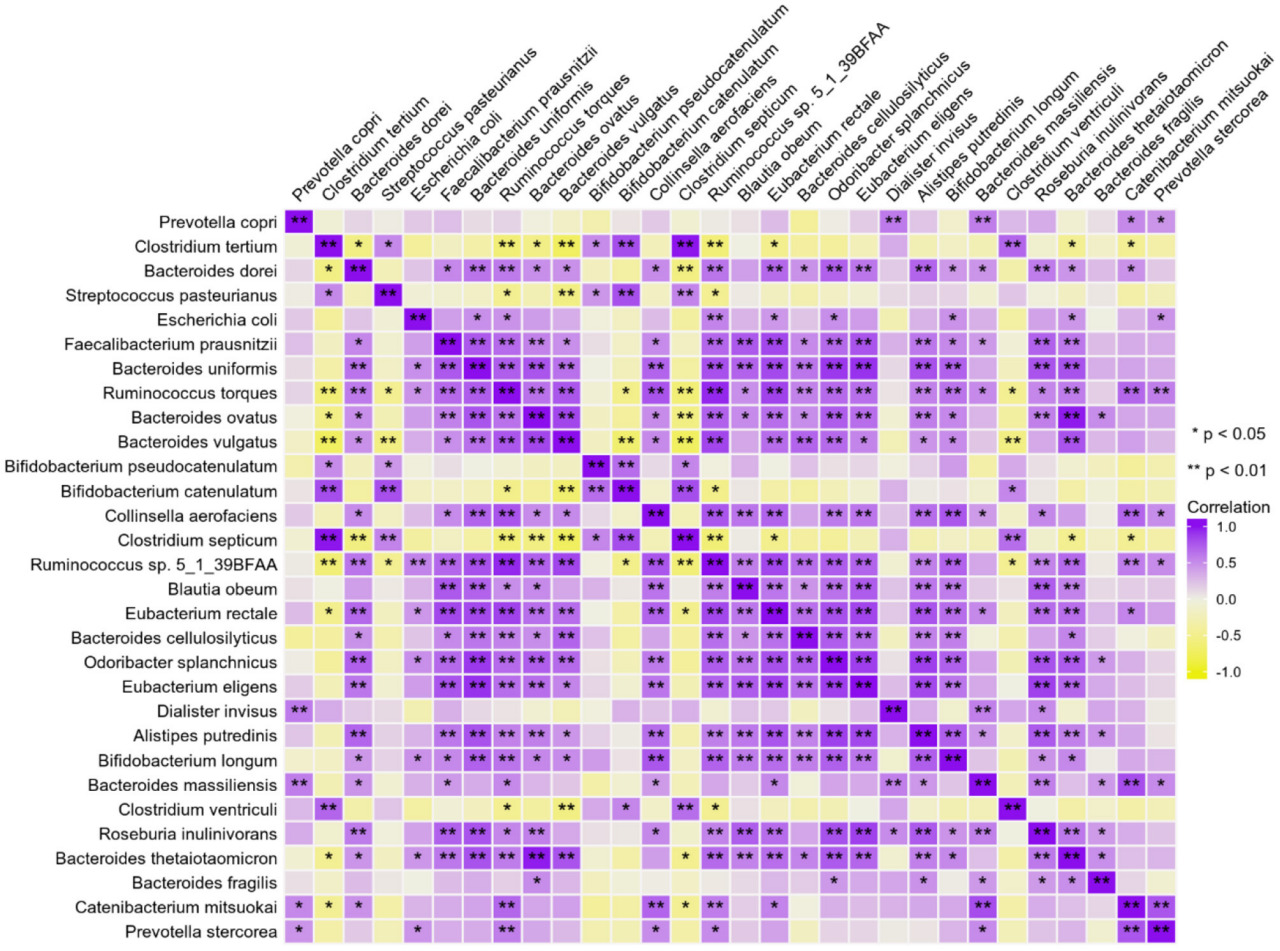
Correlation analysis of intestinal microbiota in children with T1D.

We explored different projects (PRJNA289586/PRJNA445932/PRJNA387903) from the BioProject of NCBI. The sample data of 21 patients aged 0–18 years and running successfully in the GMREPO database were analyzed. We visualized the relative abundance of the top 30 species in each sample (Figure 3). Furthermore, the Chi-square test, Spearman correlation analysis, and multifactor unconditional logistic analysis were used to analyze the original data (Figure 4). The results showed that most of the flora had a high positive correlation (correlation > 0.5) and high reliability (*P* < 0.05), whereas only a small number of flora had a negative correlation, which was similar to the Finnish sample mentioned earlier. This indicates that the abundance of intestinal microbiota is increasing and decreasing at the same time, and each intestinal microbiota may be interdependent rather than competitive.

### Analysis of Children's Type 1 Diabetes Samples Based on Age Periods

**Figure 5:**
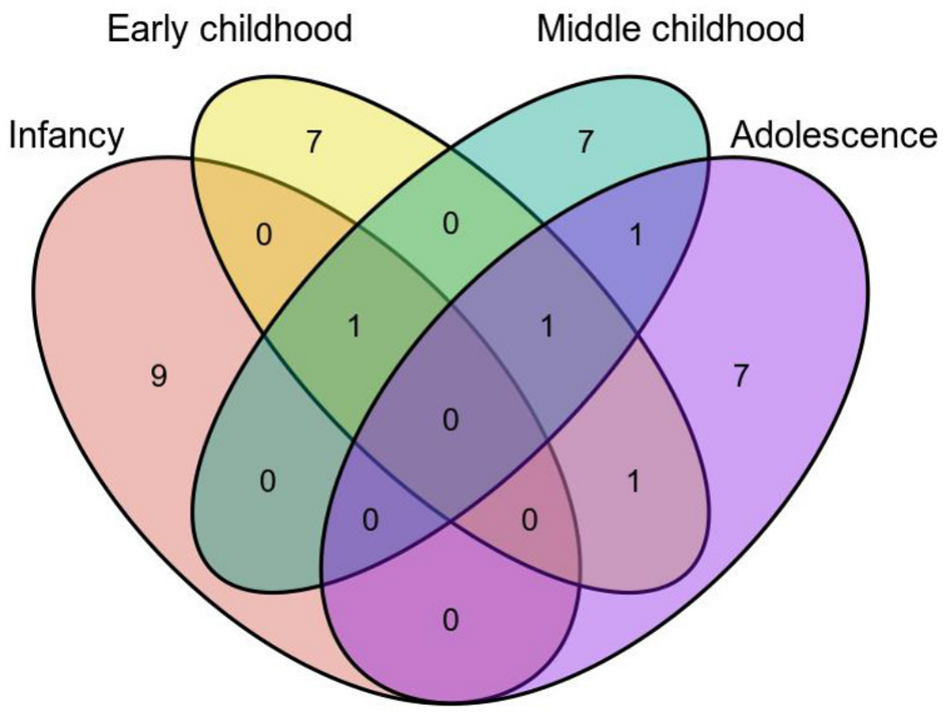
Intersection of top 30 intestinal microbiota in abundance based on age periods.

The age period of children was defined according to the World Health Organization definitions: infancy (birth to 2 years old), early childhood (3 to 8 years old), middle childhood (9 to 11 years old), and adolescence (12 to 18 years old), which divided ages into four different periods (Figure 5). The average intestinal microbiota abundance of the four age periods was calculated (Supplemental 1), and the first 30 of them were selected to draw the Wayne map for the intersection. Among them, *B. vulgatus* has a high abundance in all age periods. *Faecalibacterium prausnitzii, Blautia obeum, Odoribacter splanchnicus, Ruminococcus torques, Collinsella aerofaciens, Eubacterium rectale*, and *Alistipes putredinis* were not found only in infancy. *P. copri* have a high abundance in infancy, early childhood, and middle childhood periods.

### Difference in Intestinal Microbiota Abundance Between Male and Female

**Figure 6:**
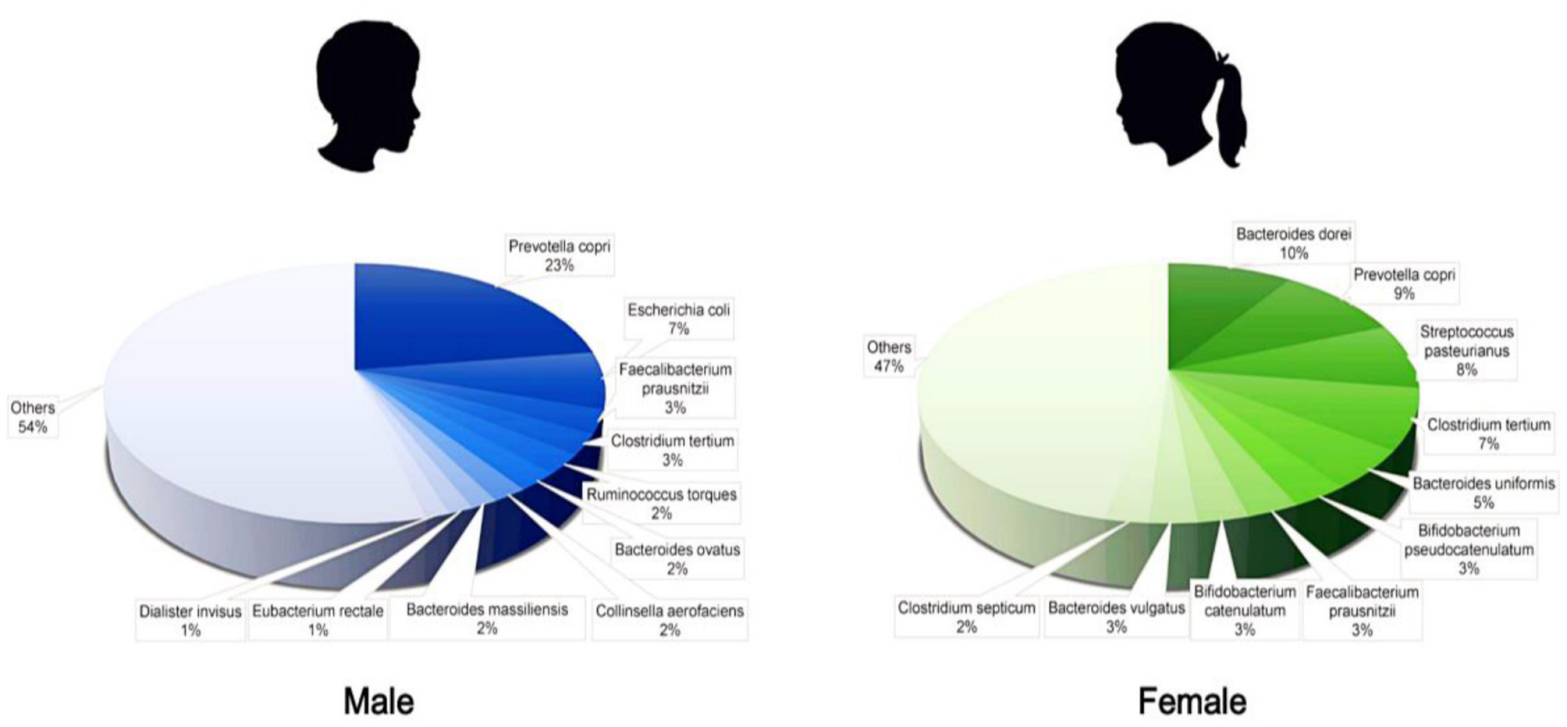
Difference of intestinal microbiota abundance between male and female.

Children were divided into female and male groups according to sex (0–18 years old). The average abundance of bacteria was calculated, and the top 10 were selected (Figure 6). We found that the abundance of each intestinal microbiota was more uniform in female samples, with the ranking from high to low as *B. dorei* 9.56%, *P. copri* 9.53%, *S. pasteurianus* 8.15%, and *C. tertium* 7.53%, whereas in male samples, *P. copri* was accounted for the largest by 22.72%.

### Visualization of Top 10 Intestinal Microbiota Between Healthy and T1D Children

**Figure 7:**
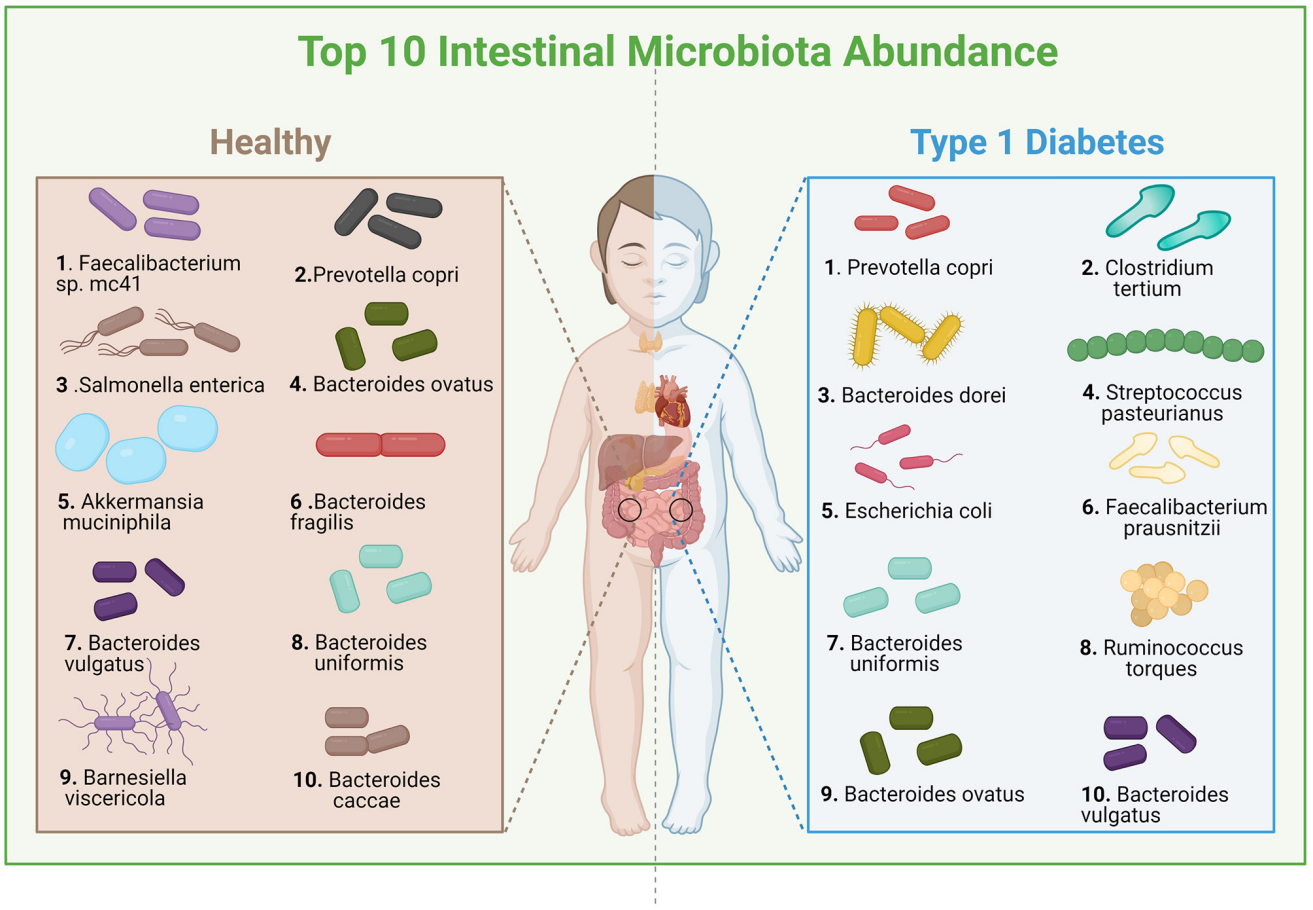
Diversity of flora abundance between healthy and T1D children.

We also obtained the sample data of healthy children aged 0–18 from GMREPO, calculated the top 10 intestinal microbiota with average relative abundance, and visually compared them with the data of children patients (Figure 7, created with *BioRender.com*). From where we could observe that there are some similarities between the intestinal microbiota of T1D children and healthy children: the abundance of *Prevotella Copri* is higher, and the species of *Faecalibacterium* are also at a higher abundance level. However, in addition to *Prevotella Copri*, the relative abundance of *Salmonella entrica* in healthy children and *Clostridium tertium* in sick children is higher, which may indicate that the disease has a greater impact on these microbiota. Moreover, *Bacteroides dorei, Faecalibacterium prausnitzii, Bacteroides uniformis*, and *Streptococcus pasteurianus* appear after infancy and are greatly affected by the disease.

## Discussion

T1D is an immune-mediated multifactorial disease characterized by auto-insulin secretion in the pancreas β cells, which are gradually destroyed, resulting in abnormal islet function, leading to a variety of problems (1–3). Genetic, epigenetic, and environmental factors determine the risk of T1D. In the past decades, the incidence rate of T1D, especially in adolescents, has been increasing. This has brought great harm to the health of adolescents. The changes in diet structure, living habits, antibiotic use, and lifestyle are closely related to the occurrence of T1D in children (3). With the deepening of the research, more and more researchers speculate that there may be an important internal relationship between the intestinal flora and the progress of diabetes. Through the study of the participants' fecal samples, scientists found that the change of intestinal flora may not only be the adverse effect of the disease but may have an important relationship with the occurrence and development of T1D in adolescents.

A number of novel research on the interaction between intestinal microbiota and T1D in children are growing to show the importance of clinical and scientific significance. Including research on children with the same HLA mutation will have different responses to bacteria in intestinal microbiota. People with T1D (usually in children) will carry HLA gene mutation, which will make individuals more susceptible to T1D, which leads to the immune response of the body to intestinal microbiota that may predict the risk of T1D (35). Other research analyzed the detailed information of thousands of molecules in cell metabolism by chemical analysis and clarified the relationship between intestinal flora metabolism and diabetes (36). Also, recently, scientists have found that microbiome targeting the gut can help prevent T1D. Dr. Emma Hamilton Williams, a researcher from the University of Queensland, came to this conclusion by comparing the differences in the intestinal microbiome between the more sensitive and the more tolerant groups (37). Meanwhile, researchers from Yale University have found direct evidence that environmental factors, such as the microbial flora in the body's intestines, may affect the development of T1D. There is a close relationship between different flora and the number of regulatory T cells. Therefore, it is possible to treat T1D by modifying intestinal microbiota (38). More interestingly, the key to metformin's regulation of blood glucose in regulating the microbes in the gut has been confirmed, which fully demonstrates that intestinal microbiota is the key to the effect of drugs in the treatment of diabetes (39). It has also been confirmed that the intestinal microbiota of children with a high genetic risk of T1D is different from that of children with a low risk of T1D, and genetic risk can affect the individual's response to environmental factors in the development of AIDs (40). In addition, some bacterial species are found in children at all but only in low or no risk children, which might hint that some species have protective effects and may be useful in the treatment of AIDs in the future.

As an organ-specific AID, T1D is triggered by both genetic and environmental factors. The importance of environmental factors in T1D, especially intestinal microbiota, has already been realized. Although further research is still needed to thoroughly explain the pathogenesis and the relationship between T1D and intestinal microbiota, the cross talk among T1D susceptibility genes, immune responses, hormones, minor food components, conventional diet, and intestinal microbiota are deeply involved. All these factors exist and play crucial roles in the pathogenesis of T1D. Nonetheless, these results must be interpreted with caution, and some limitations should be borne. First, the study mainly focused on quantitative analysis and visualization of the interaction between intestinal microbiota and T1D in children, it is hard to get or chase the intestinal microbiota of the same patient in two states at the same time, and the intestinal microbiota could transform due to different factors such as environmental change or growth and development. Therefore, the impossibility to split data according to timing with regards to disease onset, such as microbiome data before disease onset and after disease onset, should be a limitation. Furthermore, there has not been a more specific and detailed cluster discussion on the clinical situation of patients, such as mode of delivery, antibiotics in early life, and feeding type (breast-feeding *vs*. formula-feeding) (41). The impossibility to consider separately or comprehensively several risk factors could be another limitation. Exposure to a certain environment is believable to be a crucial factor that affects the intestinal microbiota of children. Also, it could also transform the environmental factors modulating immunity, then influence the pathophysiological process and disease progress. Therefore, in the follow-up study, we may supplement the description of these aspects, including collecting and analyzing more detailed medical data with comprehensive clinical management and case tracking.

## Conclusions

This study provides a new method and comprehensive perspectives for the evaluation of the interaction between intestinal microbiota and T1D in children. A set of useful information of intestinal microbiota with its internal interaction and connections has been presented, which could be a compact, immediate, and practical scientific reference for further molecular biological and clinical translational research of T1D in children.

## Data Availability Statement

The original contributions presented in the study are included in the article/Supplementary Material, further inquiries can be directed to the corresponding author/s.

## Author Contributions

JS and MZ contributed to the conceptualization of the study, formal analysis, and supervision. SX and WZ contributed to the data curation and conceptualization. MC contributed the write-up and editing of the article. JS contributed to the supervision, conceptualization, data curation, and final version write-up. All authors have read and agreed to the published version of the manuscript.

## Funding

This research was supported by the Excellent Postdoctoral Program for Innovative Talent of Hunan (2020RC2015), the Natural Science Foundation of Hunan (2020JJ5865), and China Postdoctoral Science Foundation (2020TQ0364).

## Conflict of Interest

The authors declare that the research was conducted in the absence of any commercial or financial relationships that could be construed as a potential conflict of interest.

## Publisher's Note

All claims expressed in this article are solely those of the authors and do not necessarily represent those of their affiliated organizations, or those of the publisher, the editors and the reviewers. Any product that may be evaluated in this article, or claim that may be made by its manufacturer, is not guaranteed or endorsed by the publisher.

## Supplementary Material

Supplemental 1 Analysis of children's T1D samples based on age periods (original data).

## Abbreviations

AID: autoimmune diseases
NCBI: National Center for Biotechnology Information
T1D: type 1 diabetes.
