# Supplementary material for: Quantitative Analysis and Visualization of the Interaction Between Intestinal Microbiota and Type 1 Diabetes in Children Based on Multi-Databases
Source: Front Pediatr. 2021 Dec 15;9:752250. doi: 10.3389/fped.2021.752250 (PMC8715853; doi:10.3389/fped.2021.752250)
Supplement: Supplemental 1 — Analysis of children's T1D samples based on age periods (original data). [file Data_Sheet_1.PDF]

Only infancy	Only early.childhood	Only middle.childhood	Only adolescence
Bacteroides dorei	Prevotella stercorea	Bacteroides massiliensis	Bacteroides cellulosilyticus
Streptococcus pasteurianus	Ruminococcus sp. 5_1_39BFAA	Lachnospiraceae bacterium 1_1_57FAA	Ruminococcus sp.5_1_39BFAA
Escherichia coli	Dorea longicatena	Megamonas hypermegale	Alistipes onderdonkii
Bacteroides fragilis	Sutterella wadsworthensis	Bacteroides faecis	Bifidobacterium adolescentis
Staphylococcus epidermidis	Roseburia faecis	Bacteroides sp. 2_1_22	Methanosphaera stadtmanae
Bifidobacterium merycicum	Ruminococcus callidus		Eubacterium ventriosum
Staphylococcus aureus	Bacteroides finegoldii		Anaerostipes hadrus
Unknown	Eubacterium ramulus		Clostridium saccharoperbutylacetonicum
Bifidobacterium scardovii	Lachnospiraceae bacterium 5_1_63FAA		Lactobacillus floricola
Bifidobacterium biavatii	Parabacteroides distasonis		Eubacterium hallii
Bifidobacterium breve			
Megasphaera cerevisiae			
Streptococcus equinus			
Bifidobacterium indicum			
Bifidobacterium bifidum			
Enterococcus casseliflavus			
Bifidobacterium dentium			

infancy AND early.childhood NOT middle.childhood NOT adolescence	infancy AND middle.childhood NOT early.childhood NOT adolescence	infancy AND adolescence NOT early.childhood NOT middle.childhood	early.childhood AND middle.childhood NOT infancy NOT adolescence
Bifidobacterium catenulatum	Roseburia intestinalis	Bifidobacterium pseudocatenulatum	Dialister invisus
	Streptococcus vestibularis	Barnesiella intestinihominis	Roseburia inulinivorans
	Streptococcus salivarius	Bacteroides thetaiotaomicron	Bacteroides stercoris
			Dorea formicigenerans
			Catenibacterium mitsuokai
			Coprococcus comes
			Holdemanella biformis

early.childhood AND adolescence NOT infancy NOT middle.childhood	middle.childhood AND adolescence NOT infancy NOT early.childhood	infancy AND early.childhood AND middle.childhood NOT adolescence	infancy AND early.childhood AND adolescence NOT middle.childhood
Bacteroides uniformis	Clostridium ventriculi	Prevotella copri	Bacteroides ovatus
	Clostridium beijerinckii		Bifidobacterium longum
	Eubacterium eligens		
	Parabacteroides merdae		

infancy AND middle.childhood AND adolescence NOT early.childhood	early.childhood AND middle.childhood AND adolescence NOT infancy	infancy AND early.childhood AND middle.childhood AND adolescence
Clostridium tertium	Faecalibacterium prausnitzii	Bacteroides vulgatus
Clostridium septicum	Blautia obeum	
	Odoribacter splanchnicus	
	Ruminococcus torques	
	Collinsella aerofaciens	
	Eubacterium rectale	
	Alistipes putredinis	
